# Assessing the effectiveness of a comprehensive menstrual health intervention program in Ugandan schools (MENISCUS): process evaluation of a pilot intervention study

**DOI:** 10.1186/s40814-020-00585-2

**Published:** 2020-04-24

**Authors:** Ruth Nalugya, Clare Tanton, Laura Hytti, Catherine Kansiime, Kevin Nakuya, Prossy Namirembe, Shamira Nakalema, Stella Neema, Connie Alezuyo, Saidat Namuli Musoke, Belen Torondel, Suzanna C. Francis, David A. Ross, Chris Bonell, Janet Seeley, Helen A. Weiss

**Affiliations:** 1 Medical Research Council/Uganda Virus Research Institute & London School of Hygiene & Tropical Medicine Uganda Research Unit, Plot 51-59 Nakiwogo Road, Entebbe, Uganda; 2 London School of Hygiene & Tropical Medicine, Tavistock Place, London, WC1H 9SH UK; 3 WoMena Uganda, Plot 2150 Kaduyu Close, Ntinda-Kigowa, Kampala, Uganda; 4 College of Humanities and Social Science, Makerere University, Kampala, Uganda; 5 Palm Tree Academy Uganda, Arua, Uganda; 6 Uganda Virus Research Institute, Plot 51-59 Nakiwogo Road, Entebbe, Uganda; 7 MRC Tropical Epidemiology Group, London School of Hygiene & Tropical Medicine, Keppel Street, London, WC1E 7HT UK

**Keywords:** Process evaluation, Menstrual health, Acceptability, Fidelity, Dose, Reach, School-based intervention

## Abstract

**Background:**

Poor menstrual health and hygiene (MHH) is a globally recognised public health challenge. A pilot study of an MHH intervention was conducted in two secondary schools in Entebbe, Uganda, over 9 months. The intervention included five components delivered by the implementing partner (WoMena Uganda) and the research team: (i) training teachers to implement government guidelines for puberty education, (ii) a drama skit to reduce stigma about menstruation, (iii) training in use of a menstrual kit (including re-usable pads), (iv) guidance on pain relief methods including provision of analgesics and (v) improvements to school water, sanitation and hygiene (WASH) facilities. The aim of the process evaluation was to examine implementation, context and possible causal pathways.

**Methods:**

We collected information on fidelity, dose, reach, acceptability, context and mechanisms of impact using (i) quantitative survey data collected from female and male students in year 2 of secondary school (ages 13–21; 450 at the baseline and 369 at endline); (ii) qualitative data from 40 in-depth interviews with parents, teachers and female students, and four focus group discussions with students, stratified by gender; (iii) data from unannounced visits checking on WASH facilities throughout the study; and (iv) routine data collected as part of the implementation. Quantitative data were used primarily to assess fidelity, dose and reach. Qualitative data were used primarily to assess acceptability, context and possible mechanisms.

**Results:**

Both schools received all intervention elements that were delivered by the research team and implementing partner. The drama skit, menstrual kit and pain management intervention components were delivered with fidelity. Intervention components that relied on school ownership (puberty education training and WASH improvements) were not fully delivered. Overall, the intervention was acceptable to participants. Multilevel contextual factors including schools’ social and physical environment, and family, cultural and social factors influenced the acceptability of the intervention in the school setting. The intervention components reinforced one another, as suggested in our theoretical framework.

**Conclusion:**

The intervention was feasible to deliver and acceptable to the schools and students. We propose a full-scale cluster-randomised trial to evaluate the intervention, adding a school-based MHH leadership group to address issues with school ownership.

**Trial registration:**

ClinicalTrials.gov NCT04064736. Registered August 22, 2019, retrospectively registered.

## Background

Poor menstrual health and hygiene (MHH) is a globally recognised public health challenge [1]. A recent systematic review of experiences of menstruation in LMICs highlighted the relationship between sociocultural context, resources and economic environment in impacting the lives of women and girls [2]. Poor MHH can result from inadequate education and knowledge of puberty and menstruation and from inadequate access to high-quality menstrual materials, clean water, disposal facilities and privacy for safe and effective personal hygiene [1, 3, 4]. Effective MHH interventions may lead to sustained benefits for education [5], health, productivity [1, 6] and the environment [7]. There is however, a lack of evidence of interventions to improve MHH in schools and to improve school attendance [2, 8]. Systematic reviews have identified only nine MHH completed intervention trials with health, educational or psychosocial outcomes, with inconclusive results and a high risk of bias [6, 8]. Past studies focus largely on single-component interventions, e.g. provision of pads or education only [6, 9].

We conducted formative research in four secondary schools in Entebbe sub-district, Uganda, in 2016, interviewing key stakeholders (policymakers, teachers, parents, girls and boys), and found that poor knowledge, access to materials and social support related to menstruation led to embarrassment, fear of teasing and school absenteeism [10]. The study concluded that effective school-based MHH interventions in this setting need to include (i) components to improve management of both psychosocial aspects of menstruation (education, anxiety, stigma and distress) and physical aspects (management of pain, use of appropriate materials to avoid leakage of menstrual blood and access to an appropriate MHH environment) and (ii) to include parents, boys and teachers [10]. The Menstrual Health Interventions and School Attendance in Uganda (MENISCUS) intervention was co-developed with stakeholders, to address both the psychosocial and physical aspects of menstruation and involved parents, boys and teachers in addition to girls (Table 1).

**Table 1:** MENISCUS intervention reported according to the TIDieR framework

**MENISUS intervention**	
1. **Puberty education** 2. **Drama skit** 3. **Menstrual management kit and training** 4. **Pain management** 5. **Water, sanitation and hygiene (WASH) improvements**	
**Materials and procedures**	
1. Secondary year 2 teachers from both intervention schools and other relevant teachers selected by school management received 2 days of training in how to deliver puberty education according to government guidelines using the draft National Training of Trainers Manual on Menstrual Hygiene Management compiled by the Ugandan Ministry of Education and Sports (MoES). 2. School Drama Groups with students from secondary year 1 to year 4 received two facilitated drama skit sessions (one on menstrual health and one on the drama skit process) followed by follow-up visits to drama group practices. The MHH drama was performed by the drama groups at a parental meeting at each school (an annual general meeting and a specifically organised parental meeting). The meetings were attended by teachers, parents of secondary school students and some secondary year 2 students. 3. Secondary year 2 girl and boy students were invited to participate in an MHH training session. Secondary year 2 girl students were provided with a menstrual kit consisting of a pack of AFRIpads reusable pads, a towel, soap, water bottle, knickers and menstrual calendar and an educational session on safe use and care of reusable menstrual products as well as pain management methods. Follow-up sessions were provided throughout the school year. 4. Students were provided with one voucher at baseline and throughout the study to redeem painkillers at school or a local pharmacy. All used vouchers were replaced per month. 5. WASH improvements consisted of installing locks, repairing broken doors, providing bins and toilet paper holders fixed to the wall, liquid hand washing soap and water drums.	
**Who provided**	
1. An independent educational consultant with support from WoMena Uganda staff members trained teachers from each school who are responsible for delivering puberty education in 2 days of puberty education training. The educational consultant was a professional trainer with expertise in education management. WoMena Uganda is a non-governmental organisation with expertise in programme design, monitoring and evaluation and education for menstrual health interventions. WoMena has a training team of young Ugandan menstrual health trainers (aged 20–28, with educational backgrounds in social care, nursing, education), led by the training coordinator. The puberty education sessions were supported by the training coordinator and a trainer. 2. Two facilitated drama skit sessions were delivered by the WoMena Uganda training coordinator (supported by a drama skit facilitation guide developed by WoMena Uganda). Follow-up sessions with the drama groups were led by the school drama teacher and supported by an independent drama skit consultant, who was engaged in drama skit activities during MENISCUS-1 (a previous formative study carried out in Entebbe). 3. The MHH training session was delivered by selected schoolteachers and peers who had been trained in its delivery by WoMena Uganda. Training sessions were supported by a team of six Womena Uganda trainers and the training coordinator. Follow-up sessions were provided by WoMena Uganda. 4. Painkiller vouchers could be redeemed for painkillers (paracetamol) from selected school teachers, nurse, senior women teachers and a local pharmacy. 5. WASH improvements were made by MRC/UVRI and maintained by the schools.	
**How**	
1. An education consultant and WoMena Uganda provided 2 days of group training in puberty education to teachers. 2. Two facilitated drama skit introduction sessions were held in each school by WoMena Uganda. Follow-up sessions throughout terms 3 and 1 (of year 3) were carried out by the drama skit consultant. The drama performances were arranged by the school drama teachers in collaboration with school management as part of parental meetings. 3. Menstrual management kits and training of school teachers and peers were provided in group training sessions by WoMena Uganda 4. Painkillers and vouchers were delivered to schools/students by the MRC/UVRI and LSHTM research team. 5. WASH improvements were made by MRC/UVRI and LSHTM and maintained by the schools.	
**Where**	
Staff training in puberty education was conducted outside the schools. All other activities with students and staff were conducted in schools.	
**When and how much**	
1. Two days of training in puberty education to teachers delivered in April 2017. 2. Two, 2-h introductions facilitated by WoMena Uganda were delivered in October and November 2017. A total of 21 follow-up visits to drama skit practices were carried out between November 2017 and June 2018 (10 follow-up sessions planned). Two drama skit performances were carried out in July 2018. 3. Two, 1-day training of trainers sessions delivered by WoMena Uganda in two schools to 11 female teachers, 11 female students and 2 males (drama teacher and school nurse) in May 2017. A 1-day refresher training was held in September 2017, for selected female students (5) and female teachers (6) before delivery of training to students (not planned). Training to secondary year 2 students (boys and girls) was delivered over 8 days in October and November (planned 7 days) over 17 training sessions (15 planned).	

A pilot study was then conducted in 2 secondary schools in Entebbe sub-district in 2017–2018, to determine the value and feasibility of a future cluster-randomised controlled trial to evaluate the impact of the MENISCUS intervention on secondary school attendance (primary outcome) and education, health and wellbeing outcomes [11]. The study was a longitudinal study with pre-post evaluation of a pilot intervention over 9 months. An integral part of the pilot study was a process evaluation using both quantitative and qualitative methods conducted to improve understanding of intervention implementation and possible mechanisms. The recent increase in literature on process evaluation reflects the complexity of public health interventions and the multiple ways in which comprehensive process evaluation can inform improvements in theory, intervention design and methods [12].

The aims of this paper are to (i) describe the implementation of the MENISCUS intervention in the pilot study, focusing on fidelity (quality), dose delivery (completeness), reach (participation rate) in the two intervention schools and acceptability of the intervention among intervention students, teachers and parents; (ii) describe the context of the intervention; (iii) assess possible mechanisms of action; and (iv) describe the implications for other settings.

### Intervention package

Table 1 shows the intervention components that were delivered by the implementing partner, a local non-governmental organisation (NGO) WoMena Uganda [13], in collaboration with the research team at the MRC/UVRI & LSHTM Uganda Research Unit.

### Theoretical framework for the MENISCUS intervention

**Fig. 1:**
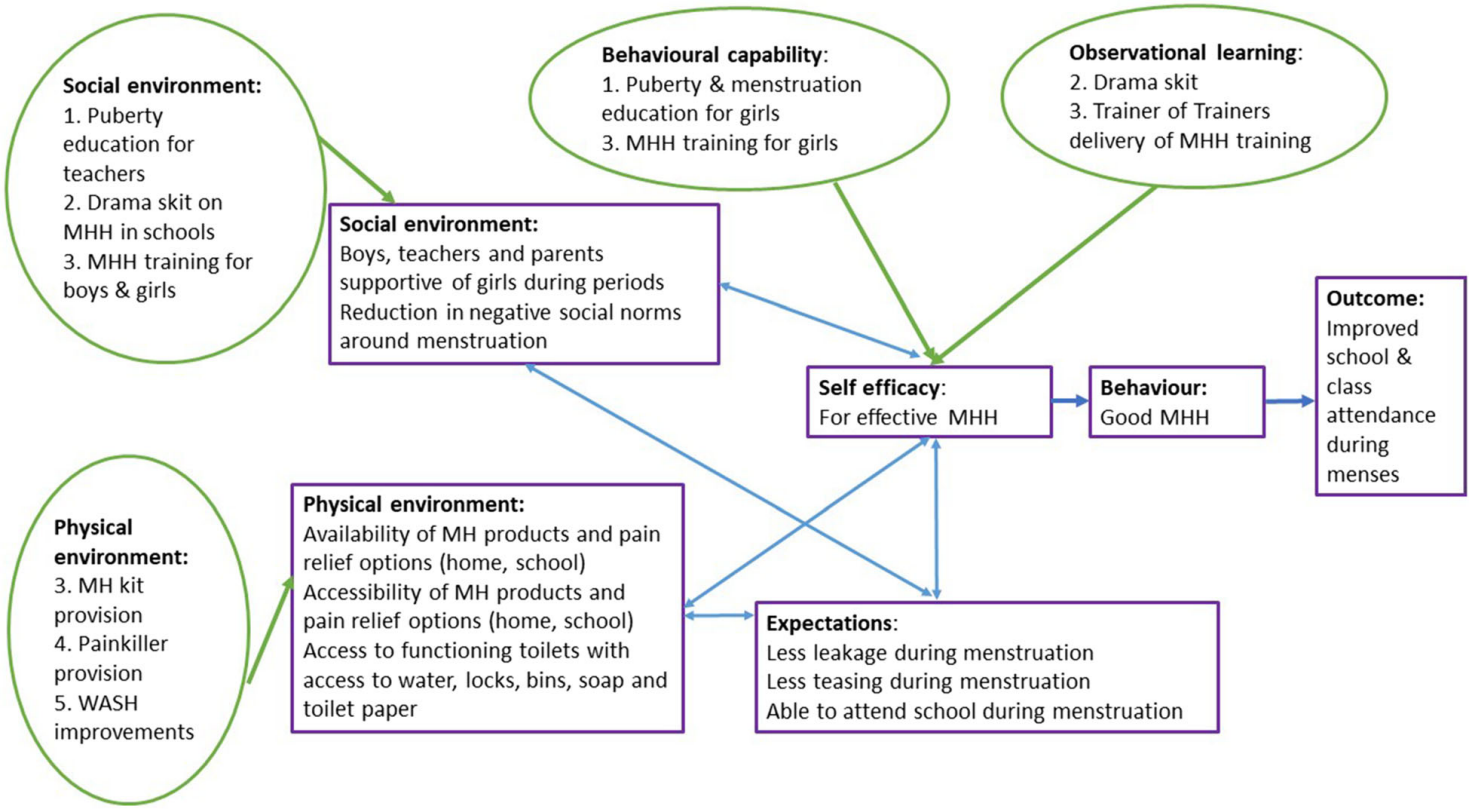
Theoretical framework for the MENISCUS intervention. Green circles show the constructs of social cognitive theory that the intervention aims to work through, with details of the intervention components addressing these in the relevant circles. These work to increase self-efficacy for effective MHH. Expectations around effective MHH are also shown. The ultimate aim is for effective MHH to lead to improved school and class attendance during menses, but this is not evaluated in this pilot trial

The MENISCUS intervention is grounded in social cognitive theory (SCT) which suggests that individuals learn by observing others. Whether an observed behaviour is learnt and enacted depends on (i) whether the individual has self-efficacy regarding the behaviour, (ii) the reinforcement an individual receives for performing the behaviour and (iii) whether the environment supports enactment [14]. The theory provides opportunities for social support through instilling expectations, self-efficacy and using observational learning and other reinforcements to achieve behaviour change. We developed a logic model of change with stakeholders (teachers, students and parents, Ministry of Education and Sports representatives, Ministry of Health, the District education officer, Makerere University and NGOs working on MHH in Uganda) during a workshop in April 2017. Aligning this logic model with the core constructs of SCT, we developed a theoretical framework for the intervention (Fig. 1) which asserts that the intervention (i) increases girls’ self-efficacy to manage their menstruation (e.g. through provision of an MHH kit and pain management options), (ii) positively reinforces girls’ learning (and also that of boys, teachers and parents) to create a more supportive MHH environment (e.g. through drama) and (iii) reinforces behaviour change through positive reinforcement and expectations (e.g. through improving WASH facilities). Figure 1 shows how the physical and social environment and individual-level factors are hypothesised to inter-relate according to SCT.

## Methods

### Study setting, design and participants

The study was conducted in Entebbe sub-District of Wakiso District, Uganda. Full details have been published previously [11]. Two low socio-economic status day schools (one government and one private) were selected for the longitudinal pre-post implementation pilot study. Eligible participants were students (male and female) in the third term of secondary year 2 (S2) at study enrolment. Of the 473 eligible students at baseline, 450 (95.1%; 232 girls and 218 boys) consented/assented. At baseline, the mean age of girls was 15.4 (SD 1.31) and the mean age of boys was 16.2 (SD 1.49). The intervention was delivered in the schools over three terms (October 2017 to August 2018).

### Process evaluation

The process evaluation follows the guidelines developed by the UK Medical Research Council (MRC) [15] including assessment of (i) implementation delivery (as compared to the intervention plan), measured through fidelity, reach, dose and acceptability; (ii) context (how external factors influence the delivery and functioning of the intervention; and (iii) possible mechanisms of impact, identifying how intervention activities, and participants’ interactions with them, might trigger change. We used both quantitative and qualitative methods to provide empirically based insights into the fidelity of implementation as well as to enable triangulation of findings [15–18].

### Data collection

Implementation measures on fidelity, dose, reach and acceptability of each of the five intervention components (Table 1) were collected in the two schools over the course of the implementation period. Data were gathered using qualitative and quantitative methods including:

i) Quantitative surveys: Information was collected by the research team in cross-sectional surveys conducted at the end of the intervention (endline) with 188 female and 181 male students (age 13–21 years) through a self-completed tablet-administered survey using OpenDataKit software within the school setting in the absence of school teachers. The survey assessed knowledge and attitudes towards menstruation, menstrual management practices, uptake and acceptability of the re-usable pads, pain management strategies and perceptions of the school water, sanitation and hygiene (WASH) facilities after the intervention was delivered.
ii) Structured observations of WASH: Unannounced WASH visits were conducted eight times in each school by the research team, using a standardised observation checklist to assess the functionality of sanitary waste bins; availability of water, soap and toilet paper; cleanliness of toilet facilities; and privacy therein. Data were recorded in a tablet-administered survey using OpenDataKit software.
iii) Voucher data: Redeemed painkiller vouchers were collected from designated school staff and pharmacies by the research team.
iv) Qualitative data: Qualitative data collection was led by a social scientist who has not been involved in implementation. Qualitative data were collected at the end of the project to assess perceptions and acceptability of the intervention component(s), and the perceived pathways to impact of the intervention. In-depth interviews (IDIs) were conducted from June to August 2018 with (i) 20 female students sampled purposively (menstruating S2 girls with different levels of reported school attendance at baseline), (ii) five teachers per school (three females and two males) identified by the head teachers as having been at the school at least 18 months; and (iii) 10 parents (three females and two males per school) who had attended the drama performance. Interviewers were the same sex as respondents. Four focus group discussions (FGDs) per school were conducted with students (two with each sex; each with 6–10 participants). Both IDIs and FGDs were conducted using semi-structured topic guides, and discussions were audio-recorded upon formal consent.
v) Routine implementation data: Routine monitoring data collected by WoMena Uganda as part of implementation were also used to provide information on fidelity, dose and reach.
vi) School reports: The schools were asked to provide a report on the delivery of the puberty training and an attendance list for the drama skit performance.

Table 2 summarises the data sources for evaluations of fidelity, dose, reach and acceptability for each intervention component.

**Table 2:** Data sources for evaluation of fidelity, dose, reach and acceptability

Component	Fidelity	Dose	Reach	Acceptability
1. Puberty education	1. Puberty training report 2. School action plans	1. Attendance list 2. Data reported by schools on puberty and menstruation training	1. Attendance list 2. Written and verbal report from schools on puberty education sessions	1. IDIs and FGDs
2. Drama skit	1. Facilitation guide checklist 2. Drama skit activity log 3. Drama skit topic checklist	1. Drama skit practice log 2. Performance report	1. Attendance lists 2. Observation log 3. Endline survey	1. IDIs and FGDs
3. MHH kit and training	1. Training attendance lists 2. Observation log 3. MHH Kit stock recorder	1. MHH kit stock recorder 2. Attendance lists 3. Observation log	1. Attendance lists 2. Follow-up report	1. IDIs and FGDs 2. Endline survey
4. Pain management	1. Voucher logbook 2. Project coordinator record 3. Stock sign off sheet 4. Observation checklist (monthly)	1. Voucher data	1. Voucher data 2. Endline survey	1. IDIs and FGDs
5. WASH improvements	1. WASH installation log 2. Unannounced observations	1. WASH checklist	1. WASH checklist 2. Unannounced observations	1. IDIs and FGDs 2. Endline survey

### Process data

Data from the sources described above and in Table 2 were used to describe implementation of the intervention. Table 3 summarises the measures of fidelity, dose and reach for each intervention component.

**Table 3:** Summary of measures for fidelity, dose delivery and reach

	Fidelity	Dose	Reach
1. **Puberty education**	Was all puberty education content delivered?	*N* of puberty training sessions delivered to teachers	*N* of teachers receiving puberty training
	Was an action plan created by the school?	*N* of puberty training sessions delivered to students	N of pupils receiving puberty education
	Was the action plan followed?		
2. **Drama skit**	Were facilitation sessions and practices carried out?	*N* of rehearsals	
	Was the performance carried out on annual parents’ day?	*N* of performances	*N* of i) parents and ii) students attending drama performance
	Delivered within expected time scales?		
	Were all core topics included?		
3. **MHH kit and training**	Was training of trainers delivered?	*N* of MHH kits distributed	*N* of girls receiving a MHH kit
	Was training to S2 students delivered by trainers?	*N* of student training sessions held	*N* of students receiving MHH training
	Were all training components covered in S2 those trained?	*N* of training components covered	
	Were all kit components included?		
	Were follow-up visits carried out?	*N* of follow-up visits	
4. **Pain management**	Did all girls receive vouchers?	*N* of vouchers given to girls?	*N* of girls redeeming painkiller vouchers
	Were procedures in place for girls to exchange?		
	Was stock of painkillers available?		
3. **WASH improvements**	Were all WASH components installed?	*N* of WASH components installed	*N* of schools receiving all basic WASH items
	Were all WASH components installed in time?		*N* of components present at follow-up
	Were the WASH improvements maintained?		Percentage of toilets functional, with lockable door and clean at final visit and percentage of visits where soap and water available

### Data analysis

Data from redeemed vouchers were entered in the Microsoft Access. Data from the endline survey, unannounced WASH observations and redeemed vouchers were analysed using Stata 15.0. Survey data were summarised to describe the proportion of students reporting receiving an MHH kit, using reusable pads during their last menstrual period, attending the drama skit and reporting an improvement in WASH facilities. Data from all unannounced WASH observations were combined to describe the proportion of visits where at least one toilet block at the school had the following: waste bins, toilet paper, a functional water drum and a functional water and soap drum. Voucher data were summarised to determine the number and proportion of girls redeeming a voucher for painkillers.

Audio-recordings for both IDIs and FGDs were transcribed verbatim, translated into English and reviewed for accuracy by the lead social scientist. Data were analysed using thematic content analysis by two social scientists (social science team leader not involved in the implementation and the senior social scientist). A coding framework was developed, with codes organised into a matrix and categorised into descriptive sub-themes and key themes which were discussed and agreed with the wider team before analysis. The two social scientists who coded the data compared their coding as they progressed, to check for consistency. We examined the acceptability inductively from qualitative data from the perspective of the intervention recipients, and we were sensitised by our a priori theoretical framework to consider component constructs such as self-efficacy to managing menstruation and attitude towards the intervention.

Data collected through routine monitoring and evaluation mechanisms (including attendance at intervention activities and observations by the research and implementation teams) were entered into an Excel database and analysed using basic descriptive analysis.

## Results

### Fidelity, dose, reach and acceptability of the intervention components

Both schools received all components delivered by the research team or the implementing partner, but with different levels of fidelity. The expected dose and reach were achieved overall (Appendix), and the intervention as a whole was considered acceptable by the schools and students.

In general, MENISCUS benefited us all. The school environment is now conducive for us girls during our menstruation because the water is available and mixed with liquid soap; the toilet doors have locks, there is privacy, a person cannot interrupt you while changing the pad. Toilet paper is available whenever you are in need and the toilets are ever clean. We were also given [reusable] pads. You can hardly find a girl in our class without a pad or with a stained dress unlike in the past. (Female aged 17, school 1)

#### Fidelity

The components of the intervention that were delivered either by the implementing partner (WoMena) or research team were delivered with fidelity. However, the intervention components that relied on school ownership (puberty trainings and WASH improvements) were not fully delivered. The 2-day puberty training for teachers was conducted as planned. The trained teachers were expected to deliver one puberty training session per term to students. However, only one of the 3 expected training sessions was delivered to students in each school. Although only one training session was reported as delivered, teachers in interviews reported making adaptations to the duration and content of puberty training, making it likely that more puberty education was delivered than reported.

The senior woman teacher drew a timetable and we had to go to particular classrooms and talk to them about puberty and menstruation. We sat and shared topics and a few of us committed ourselves to participate. However, we have not regularly followed the program as it was not included on the school timetable and most teachers are part-time teachers which was difficult to fit them in programmes outside their timetable without any incentive. (Female teacher, school 1)

A lack of time within the school schedule impacted the delivery of puberty education, drama skit presentation and MHH kit usage. For example, although students and teachers expressed appreciation for the MHH kit distribution and training, the implementation team carried out fewer follow-up visits than planned due to the perception by the schools that these visits interfered with other school activities. The drama skits were not carried out during routine music, drama and dance activities and interfered with other activities at times.

The WASH intervention was the most challenging element to implement. Although the planned WASH improvements were delivered, their maintenance (for which the schools were responsible) varied over time and by school. There was an improvement in the presence of bins in the latrine blocks and functioning water drums with soap, but these were not present during all of the unannounced check visits (Table 4). Water drums were also not always located close to the latrines, the sanitary bins were often full and toilet paper was not present in the cages provided, although it was available to students through class representatives when requested. At the final unannounced visit, all the girls’ cubicles in school 1 and half of those in school 2 had a functional lockable door and were clean.

WASH had helped us in proper sanitation and privacy during menstruation, but the population is too big which makes it difficult to maintain like refilling the water cans all the time. The prefects in charge make sure they fill every morning, but it becomes hard for them to refill between class hours. We also do not have someone assigned to clean the toilets. Students do it themselves that is why, at times, you find the place dirty and bins full of used pads because they clean once a day though this is once in a while when you find such. (Female teacher, school 1)

**Table 4:** Summary of condition of WASH facilities during eight unannounced visits

Component	Baseline (1 visit)	Follow-up (8 visits)			
	Boys and girls	Girls		Boys	
	Both schools	School 1	School 2	School 1	School 2
**Bin**	0%	6/8 (75%)	8/8 (100%)	n/a	n/a
**Toilet pape**r	0%	2/8 (25%)	0/8 (0%)	1/8 (13%)	1/8 (13%)
**Functioning water drum**	0%	7/8 (88%)	7/8 (88%)	7/8 (88%)	7/8 (88%)
**Functioning water and soap drum**	0%	6/8 (75%)	2/8 (25%)	5/8 (63%)	3/8 (38%)

Percentage of the 8 visits when the WASH component was present at at least one toilet block in the school

#### Dose

In terms of dose, the intended number of puberty education sessions to teachers was delivered, the drama skits were performed to parents in both schools, MHH training and kit distribution were carried out, vouchers for painkillers were distributed to all female study participants and all planned WASH improvements were carried out. The drama skits reached parents and students, although in one school only 35% of the girls and 50% of boys reported attending the drama skit (compared to 69% and 71% respectively in the other school). Overall, 89% of girls and boys attended the first MHH training session, and 92% of girls attended the second session (on MHH products) and received a MHH kit. All students (girls and boys) in both schools had access to WASH improvements irrespective of being part of the study, and most (81%) of girls reported improvements in WASH facilities at endline compared with baseline, which improved their comfort in menstrual management.

#### Acceptability

Opinions varied on the acceptability of the distributed menstrual pads and vouchers. Most girls (83%) reported using reusable pads during their last menstrual period, with some girls reporting that insufficient pads were provided, and girls with longer periods could not afford to supplement the reusable pads with disposable ones and would occasionally wear damp pads. Acceptability of the painkillers also varied between participants. Those with severe pain or a positive attitude towards medication before and during the study found painkillers more acceptable. Only 78 vouchers were redeemed of the 232 received by girls, although girls using the vouchers reported that the painkillers were easy to access. However, there was an increase (from 50% at baseline to 78% at endline) of girls reporting using other effective pain management such as stretching, exercise and using a hot water bottle, introduced to them in the MHH training, were effective for managing menstrual pain.

I sometimes experience stomachache and what I do, I get a bottle put warm water as MENISCUS people told us; I get a towel, place it on the stomach and start massaging. They told us to also do exercises to relieve the pain. I fear medicine that is why I rarely take it. I opt to use warm water. (Girl, aged 17 years, school 2)

Misconceptions and fears about painkillers, for example as a cause of cancer and infertility, were frequently expressed, despite this being addressed in the education session delivered to students.

You cannot take Panadol on an empty stomach. Some children come to school without eating anything; so they cannot take the Panadol when they are hungry because they say they cause ulcers or can make you not menstruate again. (Girls FGD, school 2)

The puberty education sessions were divided into a mixed-sex group and a girls-only group, and both groups reported a good learning environment that influenced participation of both boys and girls and also promoted free expression. However, boys expressed concerns about feeling unable to discuss issues openly with girls and a female facilitator present, for fear of being shamed when discussing menstruation and other issues that concerned them. They requested gender-specific sessions to increase their engagement on MHH.

### Contextual factors and mechanisms of impact

We explored contextual factors and mechanisms of impact as key interrelated elements of the process evaluation, to understand better the factors affecting the implementation process and intervention outcomes. In line with SCT, we frame our findings around individual-level self-efficacy, the school level/broader context and the physical environment.

### Contextual factors

Contextual factors influenced the implementation process and intervention outcomes at both individual and school levels. In qualitative interviews, participants explained that before the intervention, the individual and school context were characterised by poor MHH infrastructure, lack of menstrual materials, poor knowledge on MHH, stigma and lack of support from peers, teachers and parents.

As girls, we initially did not have a platform to talk about menstruation and some of our friends were not always provided with pads by their care givers…even at school, the toilets were always dirty without locks and some of us could fear to go there…male teachers would embarrass us in class and at times they would deny us passes to go back home in case of abrupt periods. (FGD girls school 2)

At the individual level, girls mentioned that meeting their MHH needs (equipping them with knowledge and physical resources) and improving the context in which they managed MHH with security, privacy and dignity made them ready to participate and more willing to use intervention products.

Given the challenges we have mentioned, I think we had no alternative but to participate. Being part of MENISCUS has changed us and our school… (FGD girls school 1)

At the school level, the appreciation and approval of the intervention by the school administration and the teaching staff enhanced their participation in, and management of, the intervention. Teachers expressed their willingness to participate in the monitoring of WASH facilities and the delivery of puberty training because it complemented their role and they perceived it as addressing the menstrual challenges that affect girls’ school attendance. However, they were limited by external factors. For example, WASH facilities in one school were also shared with community members which provided additional challenges in terms of maintaining the facilities. In addition, although improvements were made to the WASH facilities, no major structural changes were made and girls in one school reported that latrines were still very small and did not have enough light.

The cultural, social and parental contexts were also important. Teachers, parents and girls all perceived menstruation to mean that a girl had transformed into a woman, was capable of taking care of her own personal hygiene, limited to performing certain tasks during menstruation and was responsible for her sexual behaviour. For most girls, existing social beliefs and stigma perpetuated fear and prohibited them from seeking support for managing menstruation.

when I got my first periods, I feared to talk to my mother because I thought she was going to say I had had sex…I did not tell my aunt because I had heard you are not supposed to tell anyone, so I kept using papers and old clothes. (FGD girls school 1)

This provided motivation for participation in the intervention, with girls reporting a desire to learn proper menstrual practices that supported their cultural expectations and to overcome menstrual-related fears. There was also recognition by teachers and students about the importance of engaging family caregivers within MHH interventions. They mentioned that caregiver involvement broke the silence over time and triggered open discussions about menstruation and menstrual materials.

These girls have been in great fear and shock because of the cultural beliefs. You find when most of them cannot ask for a pad from their caregivers just because they are not sure of what the response might be, or because they have been told that menstruation is a personal responsibility and secret. But I think MENISCUS has helped most of us overcome this, and they will be able to discuss with their caregivers… (female teacher, school 2).

The sociocultural context also influenced the way girls in particular interacted with the intervention. Many girls expressed reservations about using painkillers because of beliefs related to infertility. Some students reported being hesitant to dispose of disposable pads in the bins provided, due to existing beliefs around disposing of pads in an open place (e.g. some said that they heard that if a dog ate the used pad, then the owner would menstruate for life). However, parents and girls reported that the intervention had addressed some of the beliefs around painkillers and disposal of pads, and this encouraged girls to seek support and continue with proper MHH practices although wider normative change was not observed.

We used to hear that pain killers like Panadol cause barrenness, cancer…but the senior women teachers and Womena team told us they were myths. They explained to us that some of our friends and parents still feel that painkillers are not good. We hear these things from those who are not part of MENISCUS… (girl aged 16, IDI)

### Possible mechanisms of impact

#### Individual level

Participants mentioned changes aligned with behavioral mechanisms in SCT (e.g. increases in self-efficacy in managing menstruation) and the theoretical framework (Fig. 1). For example, the training on puberty and menstruation, and on menstrual and pain management, led to improved knowledge, attitudes and practices toward MHH. These increased girls’ confidence in managing their menstruation.

Trainings improved our self-esteem and confidence because nowadays we are not scared of coming to school. We are comfortable coming to school during our menstruation. Before the training some of us were shy and we couldn’t stand and talk in-front of people about menstruation and puberty, but now we can. (FGD girls, school 2)

Boys and girls also reported that the content of the MHH training by the implementing partner created awareness about their own body changes, which increased acceptance of their new body image (post-pubertal), boosting their self-esteem, and encouraged boys to take part in the MHH activities like the drama skit.

MHH training involved boys, and this changed the way we used to think about menstruation and the body changes in some people. Some of us have even participated in drama about menstruation better than girls. (FGD, boys, school 1).

Tracking of the menstrual cycle was also highlighted as an important skill acquired by girls during the intervention and fostered good practices such as carrying menstrual materials at appropriate times.

It (MENISCUS) has helped me to know my days. I am free now; I prepare myself before I experience my periods […] I wash my pads before receiving menstruation and keep them in a clean place. When menstruation comes, I just pick and wear. (Girl, aged 16, school 2)

Participants highlighted the provision of hygienic and culturally acceptable menstrual materials as a strategy to improve MHH. Lack of access to menstrual materials and use of alternatives (toilet paper, old rags, sponges, handkerchiefs and stockings) was seen as a major cause of poor MHH and discomfort, leading to absenteeism or lack of concentration in school.

Some girls lack what to use during menstruation and others use toilet paper or old pieces of cloths. That’s why they decide to miss class because they do not want to get embarrassed in front of their fellow students. However, some of us got pads from MENISCUS and we stopped missing school. Reusable pads are durable and have no side effects. My parents had no problem with them. They told me they were safe. (FGD, girls, school 2)

In addition to lack of products, both male and female teachers saw menstrual pain as affecting girl’s general well-being, social relationships at school and levels of school absenteeism.

From the teachers’ narratives, the puberty training and training of trainers delivered by the implementing partner equipped them with the knowledge needed to be comfortable teaching students about MHH and puberty, using both the WoMena and national MHH educational manuals.

At the beginning of this study, they selected 20 teachers, and we went for puberty education training. It has helped us a lot because as much as we had been counselling and telling the students about puberty, we did not have a manual to follow but we were given a guide and the information so we do not find it hard to address such issues… (Female teacher, school 2)

#### Physical environment

Prior to the intervention, the physical environment undermined the girls’ capacity to look after their personal hygiene at school. Girls explained that provision of lockable doors, waste bins for used pads within toilet cubicles, waste management plans and handwashing facilities made the school environment safe and private for them allowing proper menstrual hygiene and management within the school setting.

Ever since MENISCUS came it’s easy to change our pads compared to past times. Before we did not have locks on doors but now meniscus provided locks; we can easily change our pads without interference. They also provided bins where we throw the used disposable pads. (Girls, FGD, school 1)

#### Social environment

There was evidence that the intervention improved the school social environment. Girls described psychosocial menstrual-related issues previously experienced (including shame, embarrassment, fear and anxiety) and occasions when boys had teased them for staining their clothes. They emphasized that boys’ involvement with the intervention led to improved attitudes towards menstruation.

Boys nowadays [after MENISCUS] help girls during their menstruation, when a girl’s dress gets stained a boy can give her a sweater to cover the stained part. Boys’ no longer laugh at girls during their menstruation as they used to. (Girls, FGD, school 1)

Boys also mentioned that, initially, discussing menstrual issues with girls was stigmatising for them and would cause discomfort, which came as a result of poor social interaction and their individual perceptions on menstruation. However, the intervention changed their perceptions and enabled them to be comfortable associating with menstruating girls. Girls also perceived that the training addressed social stigma by providing a platform where boys, who were perceived as the main catalysts of psychosocial stress related to menstruation, interacted with girls.

…as boys, MENISCUS has also taught us to help our female friends that might be going through their menstruation period. Personally I would not even want to sit on a chair where a girl that stained used to seat but now, I find it normal…Some times when you notice that your friend has got into her period and maybe she doesn’t know what to do or maybe has not seen and you have seen the blood stain on her dress, you can gently inform her and advise her to see the senior woman teacher. (Boys FGD, school 2)

The teachers’ willingness to take part in the puberty education training facilitated the effectiveness of the intervention. Though there was little attention given to menstrual management by teachers before the intervention, teachers attested that menstruation greatly affected school attendance and classroom concentration. In both schools, the training of trainers led to discussions with the school management and other teachers, potentially enabling whole-school support to improve MHH.

Finally, according to the school teachers, the holistic and multi-component nature of the intervention was felt to be a strength of the intervention because it addressed the multiple challenges for girls in maintaining their menstrual health within the school setting and involving the school networks (i.e. teachers, parents, boys and girls).

There is a big relationship and it is difficult to choose what was more important than the other; sanitation around our school is at times good and at times bad so it required improvement. which MENISCUS did by providing water cans and doors and locks. Though girls were given pads, they needed to wash their fingers with soap and they needed a private clean environment with water and soap to be able to maintain hygiene during their periods….I realise that as much as girls needed a lot of privacy, all of us needed education about menstruation….and for boys, you cannot exclude them from the program because if you do not educate them, they will still tease the girls and if the teasing is still there, then girls will still have a challenge to fit in school and they will miss. Like I explained before that some of them (girls) don’t attend class because of stomach pains, even when they had pads, they still needed painkillers so I can’t tell you that one of them was more important than the other. (Male teacher, school 1)

## Discussion

This process evaluation in which we employed both quantitative and qualitative methods found that the multi-component MENISCUS intervention was generally delivered with fidelity, although there were challenges with components under school ownership (puberty training and WASH maintenance) and busy school timetables affected delivery of some elements. The intervention was acceptable to the schools, students and parents. The intervention was reported to positively influence the school social environment by improving interaction between girls and boys and leading to more support from boys. Similarly, staff were supportive of the intervention, facilitating additional discussions within school management, needed for sustainability. There was some evidence of increased self-efficacy of girls for managing menstruation, through education and provision of menstrual materials, and that the intervention was successful in addressing misperceptions about painkillers and disposal of menstrual materials. However, misperceptions about painkillers were still expressed and uptake remained low, due to the use of other effective and user-friendly methods.

### Strengths and limitations

Our process evaluation enabled us to assess the extent to which the intervention was delivered as intended, as well as to understand better the context of the intervention and the mechanisms of impact. We used data from multiple quantitative and qualitative sources, which concurred, providing a detailed and robust description of the implementation process. The process evaluation had limitations. Firstly, the implementation and research teams worked closely together, possibly limiting the impartiality of the process evaluation. This was mitigated by having the qualitative research led by a social scientist who had not been involved in the implementation. Secondly, acceptability was one component of process evaluation for this study, and the literature offers little guidance on how to define or assess acceptability [19]. We assessed acceptability from the participants’ perspective, i.e. their confidence in performing the required behaviour, perceived effectiveness, the extent to which they understood the intervention and how it worked, perceived burden and affective attitude. For future assessment of intervention acceptability, acceptability will need to be defined prior the onset of the intervention [20].

### Implications for research and policy

The importance of WASH facilities to improve MHH is established [21, 22]. Our study showed that improved WASH facilities within a school setting are crucial in promoting a safe and private environment which leads to reduced stigma and potentially to reduced school absenteeism among menstruating schoolgirls. As for other WASH interventions [23, 24], this component was challenging to maintain especially, for example, where large schools needed more WASH facilities (e.g. water drums) than those provided by the project, or where the school shared toilet facilities with the general community. Another challenge was the lack of school ownership for maintaining WASH components. In response to this, we suggest modifying the intervention to ask schools to establish an MHH leadership group. These groups could include students, staff and parents and would be responsible for ensuring each intervention element is delivered, maintained and sustained by the schools. School-contextual factors affected intervention delivery, and as has been seen in school-based interventions in high-income countries, senior supportive staff were found to enhance intervention delivery through facilitating conversations with colleagues and administration [25].

Other research [26–28] has also found that increased knowledge and awareness and changes in attitudes improve girls’ education and confidence to manage their menstruation. The drama skit that was performed at the schools has not been considered in other MHH interventions, and parents, teachers and students highlighted the role they perceived it to play in enhancing information sharing. Parents, female relatives and teachers are important sources of MHH information, but they may not have sufficient knowledge or confidence to discuss menstrual issues [10, 27, 29]. Parents were trusted sources of information; thus, their involvement is essential for future MHH interventions. Further, involvement of boys positively influenced the school environment and reduced the stress expressed by girls in attending school during menstruation.

The intervention was designed to improve the context (both social and physical) in which girls menstruate. In doing so, it enabled schools to better meet the needs of menstruating girls, and by equipping girls with the information and materials needed, girls’ ability to comfortably manage their menstruation in the school environment also increased.

To ensure generalizability of the intervention, it is important to ensure that it aligns with the local socio-cultural context. This influenced how participants interacted with the intervention, with some participants expressing reservations around some components (e.g. use of painkillers) and others highlighting how they were motivated to engage with the intervention to achieve cultural expectations around menstruation. It is important to note that this may be through providing girls with the means to conceal their menstrual status as an immediate intervention. Although there was some evidence that stigmatisation of menstruation did reduce, further destigmatisation would be possible in the long term through normative change.

## Conclusion

This process evaluation found that the multi-component MENISCUS MHH intervention was feasible to deliver and acceptable to the students and the schools. Some challenges to fidelity were identified in areas where school ownership was required, which could be mitigated by a school-based MHH leadership group to address these issues. Students and staff articulated changes in response to the intervention that support the importance of the multi-component nature of the intervention in order to maximise the benefit of an MHH intervention in this population.

## Data Availability

The datasets used and/or analysed during the current study are available on request from https://datacompass.lshtm.ac.uk/.

## Appendix

**Table 5:** Summary table showing fidelity, reach and dose of the intervention components

	Fidelity		Dose		Reach	
	Measures	Results	Measures	Results	Measures	Results
**Puberty Education** 1.1.	*Was all puberty education content delivered?*	Yes	*N of puberty training session to teachers delivered*	1 (/1)	*N of teachers receiving puberty training*	S1: 10 (/10) S2: 30 (/30)
	*Was an action plan created by the school?*	Yes	*N of puberty training sessions to students delivered*	1 (/6)	*How many pupils received puberty education?*	S3: 266 from the government school
	*Was the action plan followed?*	No				
**Drama Skit** 1.1.1.	*Were facilitation sessions and practices carried out?*	Yes	*N of rehearsals*	2 (/2)		
	*Was the performance carried out on annual parents’ day?*	No	*N of performances*	2 (/2)	*N of parents attending drama performance* *N of students attending drama performance*	S1: 58 S2: 471 S1: F, 36 (69%); S1: M, 30 (71%) S2: F, 47 (34%) S2: M, 70 (50%)
	*Delivered within expected time scales?*	No				
	*Were core topics included?*	Yes				
**MH Kit** 1.1.1.1.	*Was ToT delivered?*	Yes	*N of MH kits distributed?*	215 (/232)	*No of girls receiving a MH Kit*	215 (/232)
	*Was Training to S2 students delivered by ToTs?*	Yes^a^	*N of student training sessions held*	S1: 7 (/6) S2: 10 (/9)	*N of girls and boys receiving MHM training*	404 (/450) 89% girls and boys 214 (/232) 92% girls
	*Were all training components covered in S2 training?*	Yes	*N of training components covered*	10 (/10)		
	*Were all kit components included?*	Yes^b^				
	*Were follow up visits carried out?*	Yes	*N of follow up visits*	S1: 4 (/5) S2: 4(/5)		
**Pain Management** 1.1.	*Did all girls receive vouchers?*	Yes	*N of vouchers given to girls?*	232 (/232)	*No of girls accessing analgesics*	59 (/232) 25%
	*Were procedures in place for girls to exchange?*	Yes^c^				
	*Was stock of painkillers available?*	Yes				
**WASH improvements**	*Were all WASH components installed?*	Yes	*N of WASH components installed?*	95(/95)	*N of schools receiving all basic WASH items*	100%
	*Were all WASH components installed in time?*	No				
	*Were the WASH improvements maintained?*	No			*% of toilets functional, with lockable door and clean at final visit*	S1: F, 100%; M, 33% S2: F, 50%; M, 27%
	*Was soap & water available >50% of visits*	S1: Yes S2: No				

^a^Training was delivered by ToTs but needed more support from WoMena than anticipated

^b^Ethical approval was not awarded to provide the menstrual cup

^c^There was a delay in setting up the facility for girls to exchange vouchers at a pharmacy but exchange was available within the schools

## Abbreviations

CRT: Cluster randomised trial
IDI: In-depth interview
LSHTM: London School of Hygiene and Tropical Medicine
MENISCUS: Menstrual health interventions and school attendance among Ugandans
MoES: Ministry of Education and Sports
MRC: Medical Research Council
LMP: Last menstrual period
NGO: Non-governmental organisation
PGD: Participatory group discussions
S2: Secondary two
UVRI: Uganda Virus Research Institute
WASH: Water, sanitation and hygiene
SWT: Senior woman teacher
